# The impact of specific and non-specific immunity on the ecology of *Streptococcus pneumoniae* and the implications for vaccination

**DOI:** 10.1098/rspb.2013.1939

**Published:** 2013-11-22

**Authors:** Stefan Flasche, W. John Edmunds, Elizabeth Miller, David Goldblatt, Chris Robertson, Yoon Hong Choi

**Affiliations:** 1 Immunisation, Hepatitis and Blood Safety Department, Public Health England, 61 Colindale Avenue, Colindale, London NW9 5EQ, UK; 2 Department of Mathematics and Statistics, Strathclyde University, 26 Richmond Street, Glasgow G1 1XH, UK; 3 Centre for the Mathematical Modelling of Infectious Diseases, London School of Hygiene and Tropical Medicine, Keppel Street, London WC1E 7HT, UK; 4 Institute of Child Health, University College London, 30 Guilford Street, London WC1N 1EH, UK; 5 Health Protection Scotland, 5 Cadogan Street, Glasgow G2 6QE, UK; 6 International Prevention Research Institute, 95 Cours Lafayette, Lyon 69006, France

**Keywords:** *Streptococcus pneumoniae*, carriage, competition, coexistence, immunity, vaccination

## Abstract

More than 90 capsular serotypes of *Streptococcus pneumoniae* coexist despite competing for nasopharyngeal carriage and a gradient in fitness. The underlying mechanisms for this are poorly understood and make assessment of the likely population impact of vaccination challenging. We use an individual-based simulation model to generalize widely used deterministic models for pneumococcal competition and show that in these models short-term serotype-specific and serotype non-specific immunity could constitute the mechanism governing between-host competition and coexistence. We find that non-specific immunity induces between-host competition and that serotype-specific immunity limits a type's competitive advantage and allows stable coexistence of multiple serotypes. Serotypes carried at low prevalence show high variance in carriage levels, which would result in apparent outbreaks if they were highly pathogenic. Vaccination against few serotypes can lead to elimination of the vaccine types and induces replacement by others. However, in simulations where the elimination of the targeted types is achieved only by a combination of vaccine effects and the competitive pressure of the non-vaccine types, a universal vaccine with similar-type-specific effectiveness can fail to eliminate pneumococcal carriage and offers limited herd immunity. Hence, if vaccine effects are insufficient to control the majority of serotypes at the same time, then exploiting the competitive pressure by selective vaccination can help control the most pathogenic serotypes.

## 1. Introduction

*Streptococcus pneumoniae* is a major global health problem [1], with an estimated 14 million serious cases of pneumococcal disease and 735 000 deaths annually in young children [2]. It is also a major cause of morbidity and mortality in adults, in both the developed and developing world alike. A perplexing feature of pneumococcal ecology is the co-circulation of over 90 known capsular serotypes despite their competition for the same ecological niche (the nasopharynx) [3] and differences in the duration of carriage which pose an advantage for persistence of some types [4]. With the introduction of conjugate vaccines that have proved efficacious preventing nasopharyngeal colonization [5–7] to many national vaccination schemes, this ecologic puzzle has become a public health issue, as use of the first seven-valent pneumococcal conjugate vaccine (PCV7, targeted at the seven most prevalent serotypes among invasive pneumococcal disease isolates in the USA) successfully reduced carriage and disease related to these types, but also resulted in replacement carriage and disease by non-vaccine types [8–14]. This replacement is thought to be an inevitable effect of vaccinating against subpopulations of pathogens which are competing with each other [3,15] and has stimulated the development and use of higher valency vaccines (PCV10, 13, 15 and a protein vaccine targeting all serotypes).

With the rolling out of these newer higher valency vaccines across the world [16,17], and the prospect of a valency arms-race emerging, there is an urgent need to: (i) understand the mechanisms that lead to competition between serotypes but which also permits the coexistence of many types with varying fitness, and (ii) be able to infer the likely population impact of vaccination. Mathematical models have been used for these purposes, but they have tended to model only two types and have imposed between-host competition of serotypes as an artificial factor reducing susceptibility against one serotype while colonized with another [15,18]. These approaches are likely to artificially increase coexistence [19], whereas those that incorporate between-host competition in a more meaningful way need both types to have a similar rate of secondary infections for coexistence at population level to be possible [20].

The mechanism ensuring persistence of the variety of pneumococcal serotypes in a competitive environment is largely unknown. A decreased likelihood of serotype-specific colonization following acquisition of the respective serotype (serotype-specific immunity) could support coexistence [21]. Data on natural acquired immunity against pneumococcal carriage are sparse, but longitudinal carriage studies have provided evidence for both serotype-specific and serotype non-specific immunity following acquisition [22–25]. In these, the probability for colonization with the homologous or heterologous serotype respectively was found to be reduced after previous colonization [22,23], suggesting existence of both a serotype non-specific immune response and a serotype-specific immune response against acquisition of pneumococcal colonization.

We use this information to generalize widely used deterministic approaches and present a parsimonious mechanistic model incorporating possible underlying means of immunity induced by acquisition of colonization. The model is able to reproduce many of the epidemiological and ecological features of pneumococcus carriage, i.e. coexistence, competition, epidemic and endemic patterns of disease, and serotype replacement. Moreover, we use this model to assess the impact of vaccination and show that high valency vaccines under some circumstances may not be optimal.

## 2. Methods

We generalize deterministic approaches to the aspect of serotype competition of the pneumococcus [15,18,26–28] by using an individual-based model to describe the dynamics of up to 20 serotypes, which differ in their average duration of carriage. Full details of the model are given in the electronic supplementary material, appendix. In brief, acquisition of colonization induces both a purely serotype-specific and a serotype non-specific immune response. The duration of these and the duration of carriage are assumed to follow negative binomial distributions. Immunity as considered in the model is fully protective directly after acquisition and short-lived; the effect of repeated exposure and maturation of the immune system is accounted for through an exposure-independent and serotype non-specific decreasing risk of transmission and carriage duration by age. To simulate different fitness of serotypes, the mean duration of carriage in children younger than 2 years of age was varied from eight weeks for model serotype 1 to 3.25 weeks for model serotype 20 [29,30]. For each serotype, independently, an individual can be in any of the following four states: not colonized and susceptible; not colonized and immune; colonized and susceptible; colonized and immune (for a comparison with deterministic modelling approaches, see the electronic supplementary material).

Data on two-way conversational contact patterns from a contact survey, including the UK [31] were used to calculate normalized age-specific contact rates for the age groups 0–1, 2–4, 5–9, 10–19, 20–39 and 40+ [32]. The risk of transmission per contact was chosen to decrease by age in order to mirror the decrease in prevalence with age in the UK [29] and to cause the reproduction number (the average number of secondary colonizations caused by an average individual during the course of carriage in a totally susceptible population, *R*_0_) to be in the range of 1.1 to 2.7 for the different serotypes (low force of infection (FOI) scenario), 2.2 to 5.4 (mid FOI scenario) or 4.4 to 10.9 (high FOI scenario).

We mainly considered two different vaccine scenarios: (i) a vaccine providing immunity against the two most prevalent types (bivalent vaccine), and (ii) a vaccine providing immunity against all serotypes (universal vaccine). In both cases, one dose of the vaccine was administered to children at two months of age and offered a 65% chance for a 10 year protection against the acquisition of any of the serotypes included in the respective vaccine formulation [18,33–35]. We further investigate the merit of the succession of the bivalent vaccine by the universal one for which we allowed one additional infectious individual per serotype to enter the population each month. The C++ model code can be obtained from the corresponding author on request.

## 3. Results

### (a) Competition and multiple carriage

**Figure 1.**
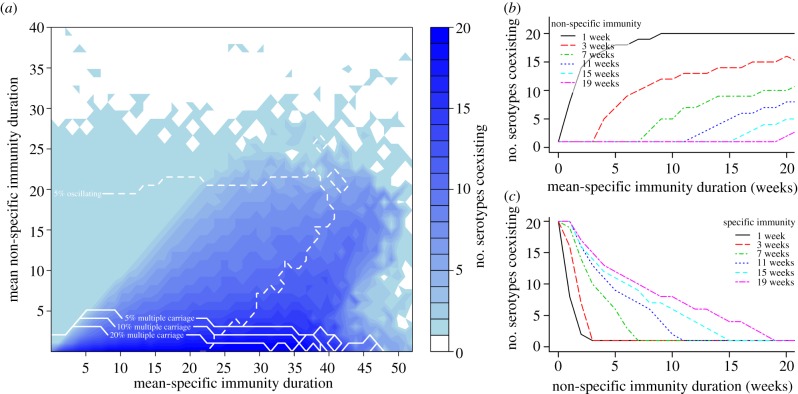
The impact of specific immunity, non-specific immunity and carriage duration on serotype coexistence in the community and the proportion of multiple carriage within the individuals. (*a*) The number of stably coexisting serotypes in the population (low FOI scenario) depending on the duration of non-specific and specific immunity. The parameter space of high fractions of the population colonized with more than one serotype at a time is indicated by white solid lines. The dashed line marks the space where at least 5% (at least one serotype, typically the most prevalent one) of all serotypes shows cyclic behaviour induced by long duration of immunity. (*b*) Positive correlation of the number of coexisting serotypes and the mean duration of specific immunity for scenarios of a fixed mean duration of non-specific immunity. (*c*) Negative correlation of the number of coexisting serotypes and the mean duration of non-specific immunity for scenarios of a fixed mean duration of specific immunity. (Online version in colour.)

The serotypes in the simulation model compete with each other through colonization-induced immunity against acquisition of all serotypes (non-specific immunity). After acquisition of a serotype and during the infectious period, this leads to a reduced pool of susceptibles available to other serotypes for onward transmission; so with increasing duration of non-specific immunity, less serotypes can coexist within the host and hence in the population owing to increasing competition (figure 1*a,c*).

### (b) Coexistence

Coexistence of multiple serotypes in the simulated community is conditional on the existence of serotype-specific immunity. If serotypes limit their own transmission only to the same extent that they limit the spread of competing serotypes, then coexistence is unlikely; in fact, if serotype-specific immunity is shorter than or equal to non-specific immunity, then one serotype will dominate, and the others will be driven to extinction. With increasing duration of serotype-specific immunity, the numbers of serotypes stably coexisting increases as type-specific immunity frees an ecological space for other serotypes (figure 1*a,b*). Coexistence of multiple serotypes within the host eases coexistence on population level but, in the presence of serotype-specific immunity, is not essential. The extent of multiple colonization of the hosts in the model is largely dependent on the duration of serotype non-specific immunity. In particular, if the duration of serotype non-specific immunity exceeds the duration of carriage, then no additional colonization is possible, and a short duration of non-specific immunity permits additional acquisition before the colonising serotype is cleared.

### (c) Variance of low prevalence serotypes

**Figure 2.**
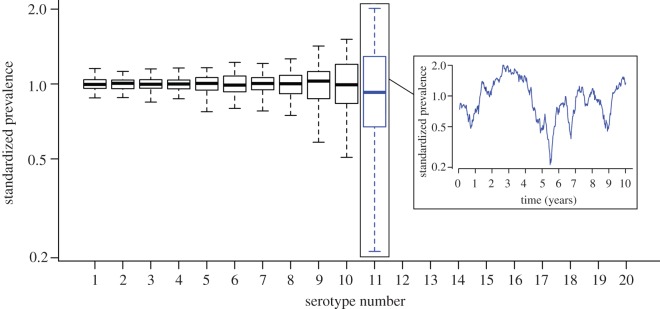
The variance of the serotypes’ standardized prevalence after the burn-in period (mid FOI scenario; mean-specific immunity duration: 18 weeks, mean non-specific immunity duration: 10 weeks, 11 serotypes were found to stably coexist in this scenario). Serotypes at the verge of extinction show relatively high deviation from their mean prevalence and peaks may stretch over several years. (Online version in colour.)

The variance of the standardized carriage prevalence increases with decreasing prevalence levels, i.e. the serotypes at the verge of extinction show high variance (figure 2). This finding is robust to the model assumptions on immunity duration, the FOI and the variance in duration of carriage (see the electronic supplementary material, figure S2). The corresponding peaks can stretch over multiple years. For those low prevalence types that have a high propensity to cause invasive disease, this effect would be amplified and picked up in the invasive pneumoccal disease surveillance as an apparent epidemic of this serotype.

### (d) Prevalence of carriage and the force of infection

Higher forces of infection generally lead to higher prevalence of carriage. However, in the presence of between-host competition, this effect is marginal, as increasing the FOI in the model leads to greater competition between serotypes, which acts to counterbalance the effect of a higher FOI on prevalence (see the electronic supplementary material, figure S3). A more important factor determining the rate of colonization is the duration of non-specific immunity. It is not, therefore, possible to conclude which of the FOI scenarios is more likely: indeed, it may well be that different settings are characterized by very different epidemiologies.

### (e) Vaccination

**Figure 3.**
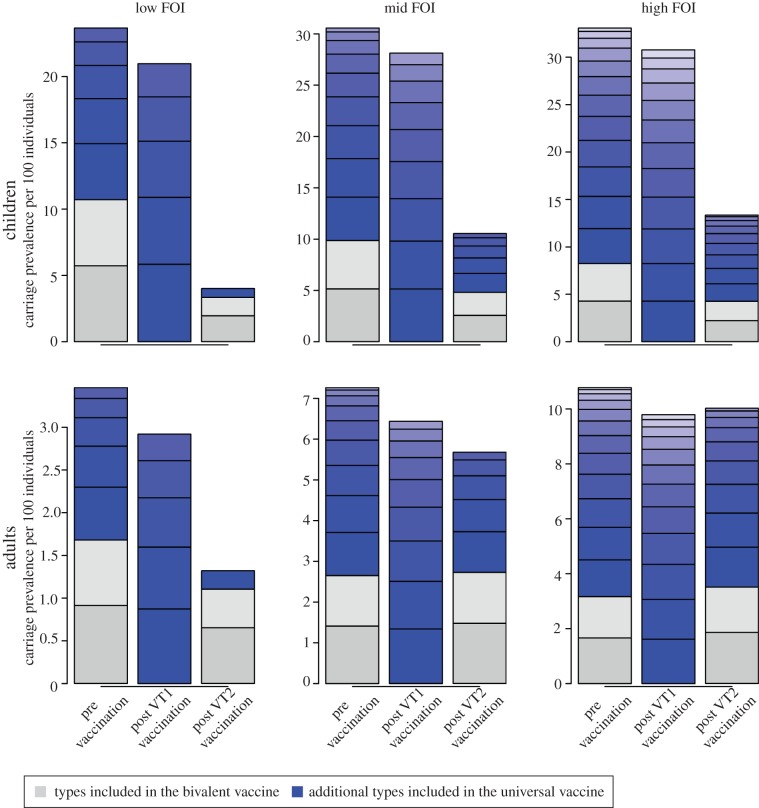
Equilibrium mean prevalence of different serotypes by age group (<10 years of age = children, others = adults) under different vaccination programmes: a bivalent vaccine targeting the two most prevalent types (VT1), and an universal vaccine (VT2) with the same efficacy per type. The duration of non-specific and specific immunity was nine and 18 weeks, respectively. (Online version in colour.)

Introducing infant vaccination against the two most prevalent serotypes into the model leads to extinction of these types in both the vaccinated and, through herd immunity, the unvaccinated individuals. Replacement with non-vaccine types in (figure 3) occurs according to the level of competition induced by serotype non-specific immunity. Paradoxically, vaccination against all serotypes—at the same efficacy and coverage—does not necessarily cause extinction of all types in the unvaccinated population and the benefits from indirect effects depend largely on the associated FOI. Indeed, under the high FOI scenario, the bivalent vaccine is more effective at reducing adult carriage than the universal vaccine. If the universal vaccine succeeds the bivalent one, re-emergence of previously controlled types can occur. This effect arises, because the vaccine effects in the model are insufficient to meet the herd immunity threshold and control all serotypes simultaneously, in particular the more prevalent ones, whereas for bivalent vaccination vaccine serotypes are controlled by a combination of vaccination effect which reduces the number of transmission events by reducing the number of susceptibles and between-host competition from the non-vaccine types (see the electronic supplementary material for further details). When the competition is lessened, through the use of a universal vaccine, and the effect of vaccination on its own is not large enough to prevent effective transmission of all types, these high prevalence (high *R*_0_) types can re-emerge (see the electronic supplementary material, figure S6).

## 4. Discussion

We present here a parsimonious mechanistic model, developed as a generalization of existing deterministic models of pneumococcal transmission, which is capable of reproducing many of the distinct features of *S. pneumoniae.* We show that the means of non-specific and specific immunity are capable of governing the patterns of between-host competition and coexistence, and that stochastic effects in low prevalent serotypes may result in apparent epidemics, if these serotypes also have a high propensity to cause invasive disease (as has been observed in several countries with serotype 1, for instance). We further show that high carriage prevalence observed in developing country settings [25] and in native populations [36] might arise from a less effective immune response (owing to malnutrition, genetic differences or other factors) rather than differences in the number of contacts alone. Moreover, we use the model to assess the impact of vaccination, and contrast a vaccine targeted at the most prevalent serotypes with a broad-based vaccine (e.g. a protein-based one), with the same level of efficacy and coverage. Paradoxically, we show that the low-valency vaccine can lead to greater indirect effects (herd immunity), if the high prevalence (most fit) serotypes are eliminated by a combination of vaccine protection and between-host competition. This benefit over the universal vaccine would be amplified in invasive pneumococcal disease if the types included in the low-valency vaccine are of high pathogenicity. Releasing the competition in the model, by using a broad valency vaccine, can further lead to a rebound in the previously controlled types.

The biological basis for protection against colonization and clearance of carriage of *S. pneumoniae* is poorly understood. However, from the patterns of pneumococcal acquisition of colonization, clearance and re-acquisition found in longitudinal carriage studies conducted over 1 and 2 years, respectively, the existence of serotype-specific and non-specific protection can be inferred [22,23]. This is reflected in the model as a short-term fully protective immunity to acquisition of colonization. With these two mechanisms, we were able to generate the specific ecological and epidemiological patterns of the pneumococcus. Short-term specific and non-specific protection might arise from a combination of both the innate and the adaptive immune response. While an activated innate response which is associated with increased macrophage or other myelomonocytic cellular activity might limit initial growth of the pneumococcus in the mucosa, the innate response is short-lived and unlikely to be the main effector for clearance of pneumococcal colonization which may persist for prolonged periods (up to five months) before clearance [37]. However, the innate response probably has the role of activating an adaptive immune response that develops to both serotype-specific capsule and conserved surface proteins that produce cross-reactive antibodies. These responses could be mediated by locally produced antibody from mucosal B cells which persist after the pneumococcus has been cleared and prevent initial growth or enhance clearance in the event of a subsequent heterologous exposure (via non-specific antibody) or homologous exposure (both non-specific and serotype-specific antibody). Although essential in murine models, the role of T-cells in clearance in the human is yet unclear [38,39]. We did not include a mechanism in the model to track colonization events over an individuals’ lifetime to infer long-term immunity which could be mediated by both capsule-specific and non-specific antigens, but chose to only include its observed effects; a serotype non-specific decline in carriage duration and the probability for transmission given contact by age. Including long-term immunity induced via past history of exposure together with a general maturation of the immune system would increase the realism of the model, but cannot be realized in a deterministic approach on which we build here.

Serotype non-specific immunity in the simulation model acts to reduce the window of opportunity for other serotypes to colonize the host and hence reduces acquisition rates. This mechanism is supported by various studies [23,27,40]. Non-specific immunity and hence competition between the serotypes could also act through increased clearance rates. However, while a general decrease in the duration of carriage by age has been identified [18,41], this could not be associated directly with exposure to the pneumococcus [27] and was reflected as such in this model. We chose a mostly data-free approach and explored the effect of various parameter assumptions on simulated rates of coexistence, carriage oscillatory patterns and multiple carriage (see the electronic supplementary material, figure S4). Most scenarios presented assumed the duration of non-specific immunity to be nine weeks. This was chosen on the basis of the rate of multiple carriage being found to be very low using current standard detection methods [8,42–45]. However, recently developed and yet to be established methods yield higher rates of multiple carriage [46]. If one accounts for that, then one would need to decrease the duration of non-specific immunity in the simulations, which would further increase the likelihood of serotype coexistence.

Other models have studied between-host competition between serotypes typically using competition as a factor limiting the FOI for additional acquisition once colonized in a deterministic two strain model [15,18,27]. While providing a useful tool to estimate the degree of competition and thereby inferring the possible effects of vaccination [47], questions about model validity in the absence of an explicit underlying mechanism have been raised [19,20]. We show that the parameter choice ensuring coexistence (serotype-specific immunity being longer than serotype non-specific immunity) in this model provides a close match to the deterministic approach most frequently used for modelling competition of two types (see the electronic supplementary material, appendix: model 4). The role of short-lived strain-transcending immunity has been previously identified to be an important determinant of the ecology of influenza by restricting the strain diversity [48]. Similarly, Zhang *et al*. [26] identified direct competition through physical presence or activated innate immune response as the most likely source of competition of the pneumococcal serotypes. In our approach, short-lived non-capsule-specific immunity induces competition and hence limits strain diversity within the host and on population level. However, we find that serotype-specific protection can counterbalance this limiting factor and lead to the vast diversity of pneumococcal serotypes.

Recently, Cobey & Lipsitch [21] published that the means of serotype-specific and serotype non-specific immunity can account for the observed diversity of the pneumococci, which supports the conclusion we reach here. In their work, serotype-specific immunity is represented as partially protective lifelong serotype-specific reduced susceptibility to carriage acquisition induced by the first clearance of colonization and an exposure driven reduction in carriage duration. The authors consider serotype non-specific immunity to be short-lived and partially protective, in particular the susceptibility for acquisition of another serotype is reduced during carriage according to the fitness rank of the most fit serotype currently carried. Similar to Cobey and Lipsitch, we do consider non-specific immunity short-lived, however fully protective (see the electronic supplementary material for details), and do not include a possible direct impact of immunity on reducing infectiousness, only indirectly through reduction of carriage duration. By contrast, we assume serotype-specific immunity to be short-lived rather than lifelong and partially protective, and attribute the reduction in duration of carriage to serotype non-specific effects by age and not exposure related. While there is evidence supporting both their approach and the one presented here, in the absence of established correlates of protection against pneumococcal carriage neither of the approaches can be overwhelmingly favoured. However, despite those differences in design, both approaches are able to reproduce observed patterns of carriage prevalence and multiple carriage as well as serotype replacement following selective vaccination, and similar conclusions are reached. Cobey and Lipsitch's work supports our finding that serotype-specific immunity may be the main driver for the coexistence of serotypes and that stochastic effects alone can account for apparent epidemics of serotypes carried at low levels and with high pathogenicity. We add to this the discussion of our approach in the context of the feasibility and possible underlying assumptions of deterministic modelling approaches and their issue of neutrality, where we find that serotype-specific immunity as modelled here could be the underlying mechanism required to ensure serotype coexistence in the model of Lipsitch [15], and also to be responsible for its non-neutrality [19]. We further explored the impact of competition on vaccination contrasting vaccines with different valencies and found that exploiting competition by selective vaccination can help with the control of some serotypes where vaccination would fail the herd immunity threshold in the absence of competition.

Long-term temporal trends and cycles have been observed in some serotypes via the surveillance of invasive pneumococcal disease. Assuming that the pathogenicity of the serotypes is unchanged, the source of these trends has to be the changing abundance of these serotypes in nasopharyngeal carriage. Interestingly, this observation has been mostly confined to infrequently carried serotypes, in particular serotype 1 [11,49,50] and has made the interpretation of the epidemiology of the pneumococcus challenging. If not accounted for, then outbreaks of highly pathogenic serotypes can substantially distort the analysis of vaccine effects, as such analyses generally attribute all changes in epidemiology to the introduction of the vaccine. For example, this was recently observed in the UK [11,51] where vaccine introduction coincided with a change in serotype 1 epidemiology. In our simulation model, we study one potential cause of this occurrence: long duration of serotype-specific immunity causes cyclic prevalence patterns with regularly occurring pronounced peaks (see the electronic supplementary material). However, this effect is most pronounced in more frequently carried types rather than the low prevalence types. Hence, to reproduce the cyclic trends being restricted to mainly low prevalence serotypes, these serotypes would need to induce a considerably longer capsule-specific protection compared with the more frequently carried serotypes. Similar to Cobey and Lipsitch, we find an additional potential cause of this epidemiologic occurrence which might interact with or amplify trends owing to serotype-specific immunity: infrequently carried serotypes are most prone to high variance in standardized prevalence solely owing to stochastic effects. Although the outbreak size can differ depending on various factors, including population size, inter-serotype differences in carriage duration and infection pressure, we consistently find that stochastic effects alone can lead to substantial differences in the prevalence for those types which are carried stably but at the verge of extinction (compare the electronic supplementary material, figure S2). This can result in peaks which spread over several years. If these low carriage prevalence but high variance serotypes are highly pathogenic (as is the case with serotype 1 [8]), then these hardly notable peaks in carriage prevalence will be amplified and picked up by surveillance systems which mainly monitor invasive pneumococcal disease.

Vaccination in the model framework with a bivalent vaccine formulation consisting of the two most prevalent serotypes and an efficacy of 65% led to extinction of the targeted types in all scenarios tested. However, the effect on overall carriage was marginal owing to serotype replacement by the untargeted types. No significant overall decline has been observed in most nasopharyngeal carriage studies assessing the impact of vaccination with the PCV7 [8,9], however, some did [43,52]. This apparent difference to our model could be due to various reasons, including the lack of power in most studies to detect this rather small decline. Although the effect on carriage was marginal, the introduction of PCV7 to national childhood vaccination programmes around the world led to substantial reduction in invasive disease, because it targeted mainly highly pathogenic serotypes. PCV10 and PCV13 are thought to have a similar effect [8].

The results on potential outcomes of vaccines with high valency presented in this manuscript are based on a highly simplified representation of pneumococcal transmission, where important aspects of pneumococcal transmission are missing, including the exclusion of partially protective immunity and reduced transmissibility as a function of previous exposure, which neglects a possible shift in carriage ranks in older age groups. A further limitation is the assumption of the model that immunity against carriage acquisition induced by vaccination is substantially longer lasting than the serotype-specific short-term immunity induced by colonization, although there is no evidence from the design of the vaccine that there should be any difference. Existing deterministic approaches implicitly make similar assumptions. Here, a model formulation of longer lasting but partially protective serotype-specific immunity could prove superior and might lead to different outcomes. Hence, our results regarding vaccination cannot be interpreted as forecasts. Rather these results illustrate a potential structural disadvantage of high valency vaccines over vaccines which only target specific pneumococcal serotypes: the loss of competitive effects induced by non-vaccine serotypes which act to further limit the transmission of the vaccine serotypes. The magnitude of this disadvantage and the associated effects are conditional on many aspects, including the strength of competition, the transmissibility of the serotypes and the vaccine effectiveness (including level of protection against carriage, coverage levels and the duration of protection), and will need to be verified in other model structures and ideally real-world scenarios. In particular, a perfectly efficacious vaccine, delivered at high coverage levels and inducing long-lasting protection is likely to eliminate transmission of all targeted serotypes, irrespective of the pneumococcus’ transmissibility and competition. However, our results suggest that a universal vaccine with similar type-specific effectiveness to the bivalent one, which is set to mirror observed vaccine effects of PCV7, may fail to eliminate pneumococcal carriage and offer limited herd immunity, because the beneficial effect of competition between types is lost. Hence, the indirect effects of high valency vaccination should be studied in detail before introduction of the vaccine to avoid possible adverse effects. These results also mean that—contrary to conventional wisdom—pursuing ever wider serotype coverage (by the use of a protein-based vaccine, for instance) may not necessarily yield increased public health benefits. Alternatively, vaccine formulations that only include those serotypes with the highest likelihood of severe disease given colonization could be an effective way to reduce the burden of disease while only causing minor disruptions to pneumococcal ecology and hence a limited probability of causing adverse effects.

The concept of pathogens competing for the same ecological niche and the implications for vaccine programmes might be applicable to other settings too. For example, quadrivalent meningococcal vaccines including the frequently carried but relatively unpathogenic serogroup Y are at risk for reducing potential competitive pressure to other serogroups, in particular serogroup B. Whether these vaccines will affect the prevalence of either the other MenB strains or other meningococcal serogroups remains to be seen. Inter-species competition has also been reported. Reduced carriage of *Staphylococcus aureus* in individuals colonized with the pneumococcus has been detected, suggesting that there may be an increase of *S. aureus* after introduction of the PCV [53–55].

We present a structurally simple model that includes serotype-specific and non-specific immunity. We find that this mechanism, which is likely to be a combination of the innate and the adaptive immune response, is sufficient to grant the key epidemiological features of the pneumococcus: coexistence, between-host competition and multiple carriage, and leads to the emergence of possible epidemic strains, as well as serotype replacement following vaccination. Furthermore, we illustrate the effect of competition on vaccination and show that selective vaccination can possibly exploit competition to enhance vaccine effects on specific serotypes where vaccine effects alone would not be sufficient to control transmission via herd immunity effects.
